# A longitudinal feature selection method identifies relevant genes to distinguish complicated injury and uncomplicated injury over time

**DOI:** 10.1186/s12911-018-0685-8

**Published:** 2018-12-07

**Authors:** Suyan Tian, Chi Wang, Howard H. Chang

**Affiliations:** 1 Division of Clinical Research, The First Hospital of Jilin University, 71Xinmin Street, Changchun, 130021 Jilin China; 2 Department of Biostatistics, Markey Cancer Center, The University of Kentucky, 800 Rose St, Lexington, KY 40536 USA; 3 Department of Biostatistics and Bioinformatics, Rollins School of Public Health, Emory University, 1518 Clifton Road NE, Atlanta, GA 30322 USA

**Keywords:** Core subset, Feature selection, Gene set analysis, Longitudinal microarray data, Significance analysis of microarray (SAM)

## Abstract

**Background:**

Feature selection and gene set analysis are of increasing interest in the field of bioinformatics. While these two approaches have been developed for different purposes, we describe how some gene set analysis methods can be utilized to conduct feature selection.

**Methods:**

We adopted a gene set analysis method, the significance analysis of microarray gene set reduction (SAMGSR) algorithm, to carry out feature selection for longitudinal gene expression data.

**Results:**

Using a real-world application and simulated data, it is demonstrated that the proposed SAMGSR extension outperforms other relevant methods. In this study, we illustrate that a gene’s expression profiles over time can be regarded as a gene set and then a suitable gene set analysis method can be utilized directly to select relevant genes associated with the phenotype of interest over time.

**Conclusions:**

We believe this work will motivate more research to bridge feature selection and gene set analysis, with the development of novel algorithms capable of carrying out feature selection for longitudinal gene expression data.

**Electronic supplementary material:**

The online version of this article (10.1186/s12911-018-0685-8) contains supplementary material, which is available to authorized users.

## Background

Currently, feature selection and pathway analysis are two bioinformatics tools that are employed with extremely high frequency. While pathway analysis aims to identify relevant pathways/gene sets associated with a phenotype of interest, feature selection mainly focuses on the identification of relevant individual genes. These two tools seem to be parallel to each other.

Nevertheless, some pathway analysis methods can be further extended to possess the ability of identify relevant individual genes. One example is the significance analysis of microarray - gene set reduction analysis (SAMGSR) method proposed by [1]. The add-on reduction step of the SAMGSR method can reduce selected gene sets into respective core subsets. Those identified core subsets provide more insight into the biological mechanisms underlying the phenotype of interest. This reduction step is a process of identifying important individual genes in nature, which motivated us to adopt the SAMGSR algorithm for feature selection [2, 3]. Here, a pathway or a gene set is defined as a set of genes that may be co-regulated/co-expressed together to impact a biological process, e.g. those GO terms in the Gene Ontology (GO) project [4] and those pathways in the KEGG database [5].

Since biological systems or processes are dynamic, researchers are interested in investigating gene expression patterns across time, in an effort to capture dynamical changes that are biologically meaningful to the systems. With the fast evolution of microarray and RNA-Seq technology, longitudinal omics experiments have become affordable and thus routine in a variety of fields. The statistical approach typically employed to analyze longitudinal omics data is to stratify the data into separate time points and then tackle them separately. This naïve strategy is inefficient given the highly dependent structure between the measures of same subject over time is erroneously ignored, leading to a huge loss of statistical power and a failure to detect incremental changes in gene expression patterns over time [6–8]. Therefore, the separate applications of cross-sectional feature selection methods (where the gene expression values were measured at a single time point) are ineffective for longitudinal microarray data [8].

On the other hand, several novel and complex methods have been proposed to specifically deal with longitudinal gene expression data [6–9]. For instance, Storey et al. [6] proposed a method designed to identify differentially expressed genes over time among different phenotypes. This method utilized spline-based models to estimate expression-versus-time curves for genes individually, and a specific software program called EDGE [10] has been developed for its implementation. This algorithm may be classified into the simplest feature selection category, namely the filter method. Since a filter method usually screens genes one by one according to a relevance score of the specific genes with the phenotype of interest, it tends to include all highly correlated features into the final model. The direct impact of this drawback is an inferior model parsimony/size (the number of genes in the final gene list) [11].

Traumatic injury with subsequent infection was a common cause of death in ancient times. Even today massive injury such as combat wounds remains life threatening [12, 13]. A large clinical study that examined the genome-wide expression patterns of blood leukocytes in the immediate post-injury period was carried out recently [14]. The authors of that study used the EDGE algorithm to characterize transcriptomic difference after severe trauma injury compared to healthy subjects, with more than 80% of genes showing significant differences between the two groups. Regarding to this unexpected “genomic storm”, we think some irrelevant genes might be included mistakenly by EDGE due to their correlations with the relevant ones.

Another primary objective of the study by [14] was to explore if different patterns of gene expression existed for two extremes of clinical recovery: the uncomplicated recovery and the complicated recovery. Likewise, the EDGE algorithm identified 2391 differentially expressed genes (DEGs) over time. Among those 2391 genes, many might be mistakenly included due to their high correlations with the true relevant genes. Given the fact one specific gene’s expression values over time are highly correlated one another and thus cluster together as a group, it is natural to regard a gene’s longitudinal expression profile as a gene set. Therefore, many gene set analysis method capable of feature selection can be utilized directly or modified correspondingly to analyze longitudinal data. In this study, we explore more deeply on the SAMGSR method [1] to investigate if it can carry out longitudinal feature selection. The microarray data in Xiao’s study [14] were used to evaluate the performance of the proposed procedure.

## Methods

### Experimental data

The raw data for the injury microarray experiment by [14] were downloaded from the Gene Expression Omnibus repository (http://www.ncbi.nlm.nih.gov/geo/). The accession number is GSE36809. This experiment was hybridized on Affymetrix HGU133 plus2 chips. Because we focus on identifying genes that present longitudinal expression change pattern between the trauma patients with complication and those without complication, only patients with uncomplicated recoveries and patients with complicated recoveries were considered here.

Based on the duration of recovery, uncomplicated recovery represents recovery within 5 days while complicated recovery includes recovery after 14 days, no recovery by 28 days, or death [14]. Thus, the potential time points for an uncomplicated recovery are days 1/2, 1, 4, 7 and 14, while those for a complicated recovery are days 1/2, 1, 4, 7,14, 21, and 28. Here, we collected 18 complicated patients with 7 full measures and 25 uncomplicated patients with 5 full measures and used the resulting dataset to train final models. Next, the data for patients with complications were truncated at 14 days in order to make comparisons with uncomplicated patients at each single time point. Lastly, we used the rest of complicated and uncomplicated patients as a test set to validate the results given by the proposed method. There were 50 uncomplicated patients and 23 complicated patients in the test set, and the time points considered in the test data were days 1/2, 1, 4, and 7 since early discharge of patients occurred before day 14. The characteristics of patients in the training set and the test set may be different since the patients in the test set were those had been discharged early from the hospitals. Of note, since different pre-processing procedures may impact the data analysis, the summary expression values of the experimental data provided by the GEO were downloaded and used directly. No pre-processing procedures were carried out.

### Statistical methods

In this subsection, we first gave an introduction to the SAMGSR algorithm, and then provided a detailed description on the proposed procedure, which is referred to as the longitudinal SAMGSR method herein.

### SAMGSR

The SAMGSR method extends the SAMGS method [15] by providing an additional reduction of significant gene sets into respective core subsets. This reduction step may approximately result in a 90% of reduction in the size of selected genes, in an effort to improve predictive performance and allow biological patterns to become more obvious. The SAMGSR method consists of two major steps [1]. The first step is the SAMGS process in which an SAMGS statistic is calculated. This statistic is the squared sum of SAM statistics over all genes within the specific gene set. Using a permutation test by perturbing phenotype labels to calculate a *p*-value for the SAMGS statistic, the statistical significance of a gene set is determined. In the second step, only those genes within significant gene sets identified by the first step are considered. The SAMGSR method reorders the genes within gene set *j* based on the magnitude of its SAM statistic and gradually partitions the entire gene set into two subsets: the reduced subset R_k_ which includes the first k genes with largest SAM statistic, and the residual subset ${\overline{R}}_k$ being the complement of R_k_ for k = 1,…, |S-1|. Here S is the size of gene set *j*. Let c_k_ be the SAMGS *p*-value of the residual subset ${\overline{R}}_k$. The optimal size of reduced set R_k_ is the smallest k such that c_k_ is larger than a pre-specified cut-off, e.g., 0.05. Conceptually, the significance level of a gene within a gene set is determined by the magnitude of its SAM statistic. It implies that if in a gene set |SAM_i_| > |SAM_j_| for genes *i* and *j*, gene *j* cannot enter the reduced subset unless gene *i* is inside the reduced subset.

### Modification to SAMGSR for longitudinal data

In the longitudinal SAMGSR method, a gene set has different meaning, namely, it refers to a gene’s expression profiles across time. Firstly, the significant genes were selected in which the original SAMGS statistic was modified to as,

1 $$ {SAMGS}_g={\sum}_{t=1}^T{d}_t^2,\kern0.5em {d}_t=\left({\overline{x}}_d(t)-{\overline{x}}_c(t)\right)/\left(s(t)+{s}_{0t}\right) $$

here, d_t_ is the SAM statistic [16] of gene g (g = 1,…,G) at time point t (*t* = 1,…, T). In the SAM statistic, ${\overline{x}}_d(t)$ and ${\overline{x}}_c(t)$ are the sample averages of gene g at time point t for the diseased and control group, respectively. Parameter s(t) is a pooled standard deviation that is estimated by pooling the expression values of all samples at the specific time point t, while s_0t_ is a small positive constant used to offset the small variability in microarray expression measurements and thus to avoid the denominator of the SAM statistic being zero. Both s(t) and s_0t_ are specific for individual time points because the variability of expression measurements may differ over time.

From the above equation, it is observed that each gene’s expression profiles over time are combined into a gene set. Namely, a gene set represents one specific gene over different time points. Our rational is that a gene’s expression values for the same individual over time are correlated, mimicking a gene set/pathway. This method first calculates the SAMGS statistics for all genes to select the relevant genes and then determines exact time point(s) where the expression values of the specific gene differ between two phenotypes.

**Fig. 1:**
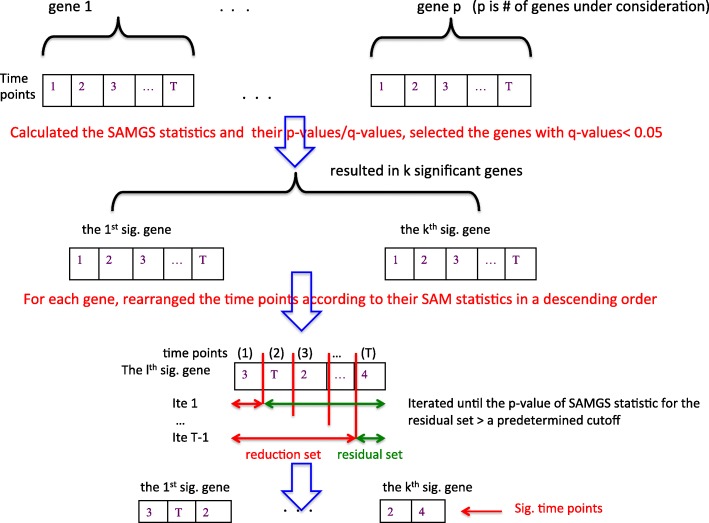
Flowchart illustrates the longitudinal SAMGSR algorithm

In the method, c_k_ is regarded as a tuning parameter. Using the sequence of 0.05, 0.1, …, 0.5, the optimal value of c_k_ corresponds to the one associated with the minimum 5-fold cross-validation (CV) error. Figure 1 elucidates graphically the longitudinal SAMGSR algorithm. Of note, the essential difference between the SAMGSR method and the longitudinal SAMGSR algorithm is what a gene set refers — while one corresponds to a set of genes and a single time point, the other contains only a single gene but many time points.

### Performance statistics

In this study, we use four metrics - Belief Confusion Metric (BCM), Area Under the Precision-Recall Curve (AUPR), Generalized Brier Score (GBS) and the misclassified error – to evaluate the performance of a classifier. Our previous study [17] described those metrics in detail. Briefly, they all range from 0 to 1. For the first two metrics the closer to 1 the better a classifier is, whereas a value of 0 is optimal for the GBS and the misclassified error.

**Fig. 2:**
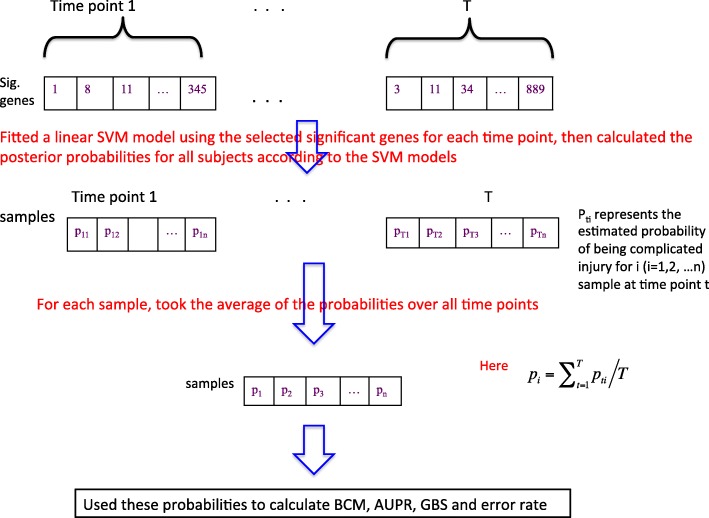
Graphical presentation illustrates how to calculate the performance statistics

Since an evaluation on individual time points using the selected statistical metrics might be unfair for the SAMGSR extension in that its tendency to identify those genes that are insignificant at isolated time points but significant jointly over time, we used the following steps to calculate overall performance statistics. Specifically, for those methods incapable of providing the final model simultaneously with the selection of relevant genes, e.g., the longitudinal SAMGSR method, a linear support vector machine (SVM) model using the selected genes was fit to estimate the β coefficients at individual time points. Then, the posterior probabilities of the samples belonging to the diseased group were calculated at each time point on the basis of the SVM models, and the averages of those probabilities over time were taken and used to obtain these performance statistics. Figure 2 shows graphically how to calculate BCM, AUPR, GBS and the error rate. For those methods that are able to provide final models, e.g., LASSO, no extra SVM fitting was needed.

### Statistical language and packages

Statistical analysis was conducted in the R language version 3.2.1 (http://www.r-project.org), and R codes for the longitudinal SAMGSR algorithm are available in an additional file (see Additional file 1).

## Results

### Injury application

Here, we applied the SAMGSR extension to the training set to build final models, whose performance was evaluated upon the test set. The respective performance statistics are provided in Table 1, from which we observed that the ability of the longitudinal SAMGSR method to discriminate between the complicated recovery and the uncomplicated recovery is the best.

**Table 1:** Performance of SAMGSR extension and other relevant algorithms on the injury data

Method	# of genes	Using 5-fold CVs				On the test set			
		Error	GBS	BCM	AUPR	Error	GBS	BCM	AUPR
L-SAMGSR^1^	97	0.442	0.268	0.515	0.576	0.356	0.230	0.535	0.622
EDGE^1^	1083	0.442	0.281	0.511	0.526	0.407	0.234	0.514	0.594
SAMGSR separately^a^	> 400	0.419	0.246	0.510	0.559	0.428	0.243	0.511	0.553
P-SVM separately	> 1000	0.488	0.281	0.477	0.454	0.441	0.244	0.511	0.560
LASSO separately	147	0.465	0.261	0.497	0.498	0.407	0.237	0.509	0.580

Note: ^a^ the posterior probabilities were calculated using an SVM classifier. Here, the cutoff for q-value in SAM-GS part is set at 0.05. # of genes represents the number of the union of individual genes selected at each time point. CV: cross-validation

In order to evaluate the predictive performance of EDGE and make a more fair comparison between EDGE and our proposed method, we conducted the EDGE analysis on the training set and evaluated its performance on the test set. The results of EDGE analysis are presented in Table 1. Compared with the performance statistics from the longitudinal SAMGSR extension, EDGE does not show any superiority. Furthermore, there were 1083 genes identified by the EDGE method. In contrast, the overall unique number of selected genes is less than one hundred with our SAMGSR extension. The superiority of the SAMGSR extension over the EDGE method in terms of model parsimony is obvious. In terms of computing time, a single run of the simple SAMGSR algorithms takes 4.03 min on a Mac Pro equipped with a 2.2 GHZ dual-core processor and 16GB RAM. A single run of EDGE takes 4.2 min if the bootstrapping method is chosen to estimate the null distribution, which is more suitable than the asymptotic normality method for this specific application because of its moderate sample size.

The other feature selection algorithms evaluated were the original SAMGSR method [1], penalized SVM [18] and LASSO [19]. Of note, these three methods were applied separately on individual time points since they are incapable of carrying out longitudinal feature selection. The results are presented in Table 1 as well. Firstly, the comparison between the SAMGSR extension and the SAMGSR separately at each time point was made. While the longitudinal SAMGSR extension accounts for the correlations among the expression values of one specific gene over time, the application of SAMGSR at each individual time point considers the membership of genes (i.e., the specific gene belongs to which gene sets, thus might interplay with other genes inside those gene sets). The results of this comparison indicate that the SAMGSR extension is superior to the separate SAMGSR procedure. Incorporation of the pathway information inside gene sets, the clusters of genes that might be potentially co-expressed/co-regulated together, did not result in the separate SAMGSR method having substantially superior performance. One possible explanation relates to the quality of the pathway database itself. The canonically curated databases on pathways/gene sets are biased toward well-studied diseases such as cancers, and with few work has investigated on traumatic injury using gene expression profiles, the gene-to-gene interaction information contained inside those curated pathways may have no or limited meaning for injury. Therefore, we conclude that the consideration of coordinated effects existing among one gene’s expression profiles over time results in a better predictive performance.

**Fig. 3:**
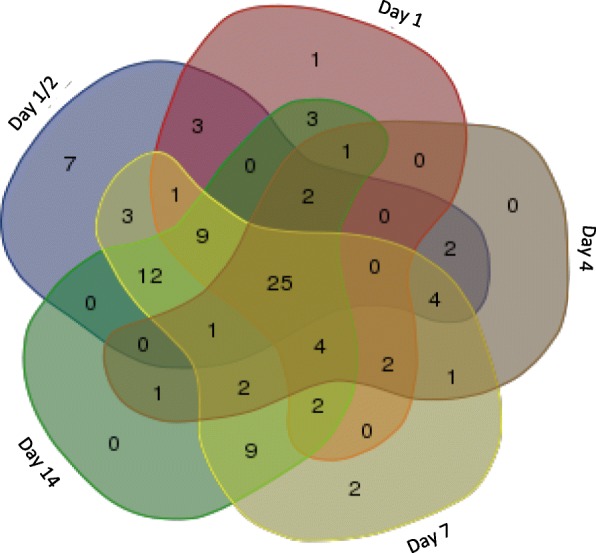
Venn-diagram illustrates how selected genes by the longitudinal SAMGSR method overlap at different time points

Secondly, we compared the longitudinal SAMGSR algorithm with well-known conventional feature selection algorithms, namely, LASSO and penalized SVM separately at each time point. In these comparisons, we observed that the SAMGSR extension has at least comparable predictive performance but being superior in terms of overall model parsimony. For example, the longitudinal SAMGSR method identifies 97 unique genes while LASSO selects 147 genes. Moreover, we observed that in the 97- gene signature identified by longitudinal SAMGSR, there is a substantial proportion of overlaps for all 5 time points together (25.77%), while the number of genes being significant only at one specific time point is one half of this number (Fig. 3). Again, this highlights the ability of our SAMGSR extension to identify genes that present mild but concordant change over time points between two different phenotypes. In contrast, though at each individual time point LASSO selects the smallest number of genes, there is almost no overlap among those genes, resulting in a 147-gene list.

Regarding computing time, LASSO is the most efficient with one single run only taking less than 1 s since the implementation of LASSO in the R glmnet package [20] calls upon Fortune language and utilizes a fast-updating strategy. These two factors make substantial contributions to the time efficiency of LASSO. On the other hand, penalized SVM is the least time efficient one, which is unsurprising since the cross-validation process is automatically a part of model construction inside the penalized SVM modeling.

### Simulations

In order to explore the properties of the SAMGSR extension, we used the observed expression values from the injury application to design two sets of simulations. First, we chose two causal genes – F13A1 and GSTM1 – and then randomly selected 998 genes from the remaining genes as noises that are irrelevant. Denote the expression value of gene *i* (F13A1 or GSTM1) at time *j* (j = 1,…, 5) as X_i.j_, the following logit function was used to obtain the probability for sample *i* (*i* = 1, …43) experiencing a complicated injury, i.e., p_i_,

$$ \log {it}_{c/u}=0.18{X}_{F13A1.1}+0.57{X}_{F13A1.2}+0.29{X}_{F13A1.3}+0.41{X}_{F13A1.4}+1.02{X}_{GSTM1.3} $$

Here, p_i_ = exp.(logit_i_)/(1 + exp.(logit_i_)). Then a random variable Y_i_ that follows a Bernoulli distribution with the parameter of p_i_ was simulated, it has two possible values with 1 indicating the sample belongs to the complicated injury group and 0 for the uncomplicated injury group. Notably, we considered one gene (i.e., F13A1) whose significance arises from its joint association accumulated from time point 1 to time point 4 and the other (i.e., GSTM1) whose association with the outcome is only at the third point time in this simulation.

The aim of this simulation was to illustrate the advantage possessed by the SAMGSR extension, namely, by incorporating the accumulated effect of genes over time, it can recognize genes with a coordinated change accumulating over time but only mild or moderate change at each time point.

In the second simulation, we chose two genes – COX4I2 and RP9 as the relevant genes. The logit function was,

$$ \log {it}_{c/u}=0.56{X}_{COX4I2.1}-0.91{X}_{RP9.5} $$

In this simulation, both genes were associated with the outcome at a single respective time point but in opposite directions. For both simulation settings, 50 replicates/50 datasets were generated. The frequencies of each causal gene being selected at each time point are given in Table 2.

**Table 2:** Performance of the longitudinal SAMGSR on simulated data

		Time 1	Time 2	Time 3	Time 4	Time 5
	# of genes	19.84	19.14	13.68	9.30	11.00
Simulation 1	F13A1 (%)	72	100	100	92	68
(Ave. # 32.06)	GSTM1 (%)	0	0	62	22	0
	# of genes	182.38	56.18	35.44	30.94	123.84
Simulation 2	COX4I2 (%)	96	0	0	0	4
(Ave. # 291.98)	RP9 (%)	10	4	4	6	96

Note: Ave. # represents the average number of the union of individual genes selected at each time point over 50 simulated datasets; # of genes represents the average number of individual genes selected at the specific time point over 50 simulated datasets; % represents the percentage of the corresponding true causal gene being selected by the algorithm over 50 simulated datasets

Although in the second simulation the number of relevant time points was less than that in the first one, the number of selected genes by the longitudinal SAMGSR algorithm was dramatically larger in the second simulation. This might be because the relevant genes in the second simulation were highly correlated with other genes compared to the first simulation. The highly correlated structure between relevant features and irrelevant ones produced a large number of redundant features that the SAMGSR extension cannot exclude. To our best knowledge, however, many feature selection algorithms, especially the filter methods [11], suffer from this drawback. As illustrated in our previous work [21, 22], an additional reduction step using a statistical method such as bagging [23] may alleviate this problem.

## Discussion

In the injury application, only complicated patients with five measurements, and uncomplicated patients with seven time points were included in the training set. Then patients discharged from the hospital earlier (thus having later measurements missing) were used to verify the resulting models. Similar to SAMGSR, our proposed extension has no difficulty to deal with missing values. Therefore, the proposed algorithm does not require this restriction. In this study, we imposed this restriction to simplify our data analysis.

The SAMGSR extension incorporates the correlated structure of expression’s profiles over time into the framework of gene sets/pathways, and is more likely to identify genes with aggregated effects over time, while their effect size at individual time points may be insignificant. These genes are usually overlooked by the implementation of a conventional feature selection method at individual time points. Using a real-world application, we showed that the longitudinal SAMGSR method is superior to other relevant algorithms.

## Conclusions

In this article, we applied the SAMGSR algorithm to carry out feature selection for longitudinal gene expression profiles. To the best of our knowledge, this study is one of few efforts to explore the modification of suitable pathway analysis methods to execute feature selection for longitudinal gene expression data, such an application may save time of developing a new feature selection algorithm that can tackle longitudinal data from scratch. As shown by two simulations, this extension has a big drawback, namely, including many redundant irrelevant genes in the final lists. Nevertheless, we believe this work will pave the way for more research to bridge feature selection and gene set analysis with the development of novel algorithms to tackle longitudinal omics data. For instance, many gene set analysis methods such as [24] may be applied directly or modified correspondingly to identify real ‘driving’ features that characterize the phenotype of interest better.

## Additional file

Additional file 1: The R codes of the longitudinal SAMGSR method. (TXT 7 kb)

## Abbreviations

AUPR: Area under the precision-recall curve
BCM: Belief confusion metric
CV: Cross-validation
DEG: Differentially expressed gene
GBS: Generalized brier score
GEO: Gene Expression Omnibus
GO: Gene ontology
LASSO: Least absolute shrinkage and selection operator
SAM: Significance analysis of microarray
SAMGS: Significance analysis of microarray-gene set
SAMGSR: Significance analysis of microarray-gene set reduction
SVM: Support vector machine
